# Prognostic Value of the Controlling Nutritional Status (CONUT) Score in Patients Who Underwent Cytoreductive Surgery Combined with Hyperthermic Intraperitoneal Chemotherapy

**DOI:** 10.3390/cancers16152727

**Published:** 2024-07-31

**Authors:** Myung Il Bae, Hyunjoo Jung, Eun Jung Park, Young Lan Kwak, Young Song

**Affiliations:** 1 Department of Anesthesiology and Pain Medicine, Yonsei University College of Medicine, Seoul 03722, Republic of Korea; bmi87@yuhs.ac (M.I.B.);; 2 Department of Surgery, Asan Medical Center, University of Ulsan College of Medicine, Seoul 05505, Republic of Korea; 3 Anesthesia and Pain Research Institute, Yonsei University College of Medicine, Seoul 03722, Republic of Korea

**Keywords:** CONUT, cytoreductive surgery, hyperthermic intraperitoneal chemotherapy, mortality

## Abstract

**Simple Summary:**

Nutritional status is closely related to the outcomes of cytoreductive surgery combined with hyperthermic intraperitoneal chemotherapy (CRS-HIPEC). However, the prognostic value of the Controlling Nutritional Status (CONUT) score in CRS-HIPEC has not yet been investigated. This study evaluated the predictive power of the CONUT score for mortality and postoperative complications after CRS-HIPEC. We found that patients with high CONUT score exhibited significantly higher 1-year mortality and postoperative complication rates than those with low CONUT score. Notably, a high CONUT score was identified as an independent risk factor for 1-year mortality and postoperative complications. These results suggest the potential of the CONUT score as a robust risk stratification tool for identifying high-risk patients within the CRS-HIPEC surgical demographic.

**Abstract:**

The Controlling Nutritional Status (CONUT) score is a novel nutritional index that integrates the serum albumin level, peripheral blood lymphocyte count, and total cholesterol level. This retrospective study explores its prognostic significance in patients undergoing cytoreductive surgery combined with hyperthermic intraperitoneal chemotherapy (CRS-HIPEC). We included 436 patients who underwent CRS-HIPEC, categorized into low (0–3) and high (4–12) CONUT score groups, and performed logistic regression analysis to predict one-year mortality and postoperative morbidity. Our findings revealed that high CONUT scores correlate with increased one-year mortality (47.1% vs. 20.3%, *p* < 0.001) and morbidity (39.2% vs. 18.2%, *p* < 0.001) compared to low CONUT scores. Multivariable regression analysis confirmed high CONUT scores as independent predictors of one-year mortality (odds ratio: 2.253, 95% CI: 1.014–5.005, *p* = 0.046) and postoperative morbidity (odds ratio: 2.201, 95% CI: 1.066–4.547, *p* = 0.033). These results underscore the CONUT score’s effectiveness as an independent marker for evaluating risks associated with CRS-HIPEC, emphasizing its potential to improve risk stratification.

## 1. Introduction

Cytoreductive surgery combined with hyperthermic intraperitoneal chemotherapy (CRS-HIPEC) reportedly improves the survival rates of patients with peritoneal carcinomatosis, which was previously regarded as an incurable condition [1,2]. Nevertheless, CRS-HIPEC remains a high-risk procedure associated with notable mortality and morbidity rates, with a procedure-related mortality rate of 4.8% and a complication rate of 21.5% [3]. Consequently, risk stratification and careful patient selection are crucial to minimize risks and optimize outcomes.

Nutritional status is a pivotal factor in determining eligibility for CRS-HIPEC [4,5,6]. In particular, malnutrition, which is prevalent among patients with peritoneal carcinomatosis, is associated with a higher mortality rate and increased postoperative complications [7,8,9,10]. Previous nutritional assessments, such as the Subjective Global Assessment (SGA) and serum albumin levels, have been criticized for their potential inaccuracies due to confounding factors and observer bias [11,12,13,14]. The Controlling Nutritional Status (CONUT) score represents a newer nutritional index that integrates the lymphocyte count along with the albumin and cholesterol levels, allowing for a more comprehensive assessment [15,16,17]. The prognostic value of the CONUT score in various major surgeries has been confirmed by previous studies [18,19], and a recent meta-analysis has reinforced the correlation between the CONUT score and prognosis of patients with cancer [20,21,22,23]. Given the crucial role of nutritional and inflammatory statuses in the outcomes of CRS-HIPEC [7,8,9,10,24,25], the CONUT score is anticipated to be a significant prognostic indicator in this patient group. Nonetheless, the predictive utility of the CONUT score in CRS-HIPEC has not yet been explored. Therefore, the current study aimed to evaluate the prognostic value of the CONUT score in patients who underwent CRS-HIPEC by retrospectively analyzing its capability for predicting mortality and postoperative complications.

## 2. Materials and Methods

This retrospective study was conducted at Gangnam Severance Hospital in Seoul, Republic of Korea, and adhered strictly to the ethical standards of the Declaration of Helsinki. Approval was granted by the Institutional Review Board of Yonsei University Gangnam Severance Hospital (approval number: 3-2024-0018, approval date: 8 March 2024). Due to its retrospective nature, the requirement to obtain informed consent was exempted.

### 2.1. Study Participants

The study cohort comprised patients who underwent elective CRS-HIPEC at Gangnam Severance Hospital from November 2014 to December 2021. Patients who were below 19 years of age or had incomplete medical records were excluded from this study.

### 2.2. Surgical Procedure

CRS-HIPEC was performed according to our institutional standardized protocol, as previously described [26]. Briefly, the surgical approach involved cytoreductive procedures, including resection of metastatic organs and peritonectomy using the Sugarbaker technique. The HIPEC phase was executed immediately after cytoreduction, which involved infusion of 35 mg/m^2^ mitomycin C in 3 L hypertonic solution (DIANEAL peritoneal dialysis solution with 1.5% dextrose; Boxter Healthcare Corp., Deerfield, IL, USA). Mitomycin C was administered in doses of 17.5 mg/m^2^ and 8.8 mg/m^2^ at 30 and 60 min, respectively. The solution was circulated by a HIPEC pump (Belmont Hyperthermia Pump, Belmont Medical Technologies, Billerica, MA, USA) at a rate of 800–1000 mL/min, maintaining a temperature of 42–43 °C for 90 min. Bowel anastomosis was performed after HIPEC.

### 2.3. Data Collection

We collected demographic and clinical information, including age, gender, body mass index (BMI), and American Society of Anesthesiologists (ASA) physical status classification. Details regarding the primary tumor sites and preoperative comorbidities like hypertension, diabetes, coronary artery disease, chronic obstructive pulmonary disease, hepatitis, chronic kidney disease, and anemia were also recorded. Preoperative laboratory results regarding the lymphocyte count and albumin, cholesterol, glucose, creatinine, and hemoglobin levels were obtained. The CONUT score, Prognostic Nutritional Index (PNI), and Geriatric Nutritional Risk Index (GNRI) were calculated based on the obtained data, following the methodologies outlined in previous studies [17,27,28].

Intraoperative data included the surgical duration, fluid administration, urine output, estimated blood loss, and amount of transfused packed red blood cells. Peritoneal cancer index (PCI) score and completeness of cytoreduction (CC) score were determined intraoperatively, with the PCI score ranging from 0 to 39 across 13 regions and the CC score ranging from 0 to 3 based on the residual tumor size [29].

Postoperative data included the length of stay in the intensive care unit and hospital, reoperation within 30 days, in-hospital mortality, 1-year mortality, overall mortality, and complications (e.g., anastomotic leakage, abscess, gastrointestinal obstruction, fistula, surgical site infection, ascites, urinary tract infection, pneumonia, re-intubation, cardiac complications, and acute kidney injury). Acute kidney injury was defined based on the Kidney Disease: Improving Global Outcomes guidelines [30]. The dates of surgery, death, and last follow-up were recorded.

### 2.4. CONUT Score Calculation

The CONUT score was determined by evaluating the serum albumin and cholesterol levels and lymphocyte count, consistent with established methodologies [17]. The score ranged from 0 to 12, with a higher score indicating poorer nutritional status. The patients were divided into two groups based on previous studies [31,32,33,34,35]—namely, (i) a low CONUT score group, which included patients with a CONUT score of 0–3, and (ii) a high CONUT score group, which consisted of patients with a CONUT score of 4–12. There are various cut-off values for the CONUT score in cancer patients, but many studies on cancer surgery have suggested 4 as the appropriate cut-off value [31,32,33,34,35].

### 2.5. Study Endpoints

For this study, the primary endpoint was 1-year mortality after CRS-HIPEC, whereas the secondary endpoints were overall mortality and a composite measure of postoperative morbidity. This morbidity composite included anastomotic leakage, abscess, gastrointestinal obstruction, fistula, surgical site infection, ascites, urinary tract infection, pneumonia, reintubation, cardiac complications, and acute kidney injury.

### 2.6. Statistical Analysis

Statistical analysis was conducted with IBM SPSS Statistics for Windows, version 23 (IBM Corp., Armonk, NY, USA), and MedCalc software, version 22.014 (MedCalc Software, Ostend, Belgium). We used the Kolmogorov–Smirnov test to check the normality of continuous data. Variables following normal distribution were analyzed using the independent *t*-test and presented as means ± standard deviations. Those not normally distributed were shown as medians with interquartile ranges (IQRs) and analyzed via the Mann–Whitney U test. Categorical variables were expressed as percentages and analyzed with the chi-squared or Fisher’s exact test.

Logistic regression analysis was conducted for 1-year mortality. Initially, all variables were compared between the 1-year mortality and non-mortality groups using the aforementioned tests, selecting variables with *p* < 0.20. Univariable logistic regression was applied to the selected variables to calculate the odds ratios (ORs) and 95% confidence intervals (CIs). Significant variables from the univariable analysis (*p* < 0.05) were further analyzed using multivariable logistic regression. A pathological variable, which comprises four groups (colorectal cancer, gastric cancer, appendiceal cancer/pseudomyxoma peritonei, and others) based on the disparities identified in Supplementary Table S1, was utilized in the regression analysis. Since CONUT score and preoperative anemia were included in the regression analysis, components of the CONUT and hemoglobin were excluded from the model. Multicollinearity was checked through the variance inflation factor. In addition, we categorized the patients into subgroups based on cancer type, PCI score, and comorbidities, and performed logistic regression analysis to predict 1-year mortality.

Cox regression analysis was performed to investigate risk factors for overall mortality. Univariable Cox regression was applied to each variable, and the results were expressed as hazard ratios (HRs) and 95% CIs. Variables with significant HRs (*p* < 0.05) in the univariable analysis were subjected to a multivariable Cox regression analysis.

The methodology for investigating the risk factors for the morbidity composite mirrored that employed for 1-year mortality, with both univariable and multivariable logistic regression analyses being performed. Overall survival probability was illustrated using Kaplan–Meier curves, and between-group differences were examined using the log-rank test. Statistical significance was set at *p* < 0.05.

## 3. Results

**Figure 1:**
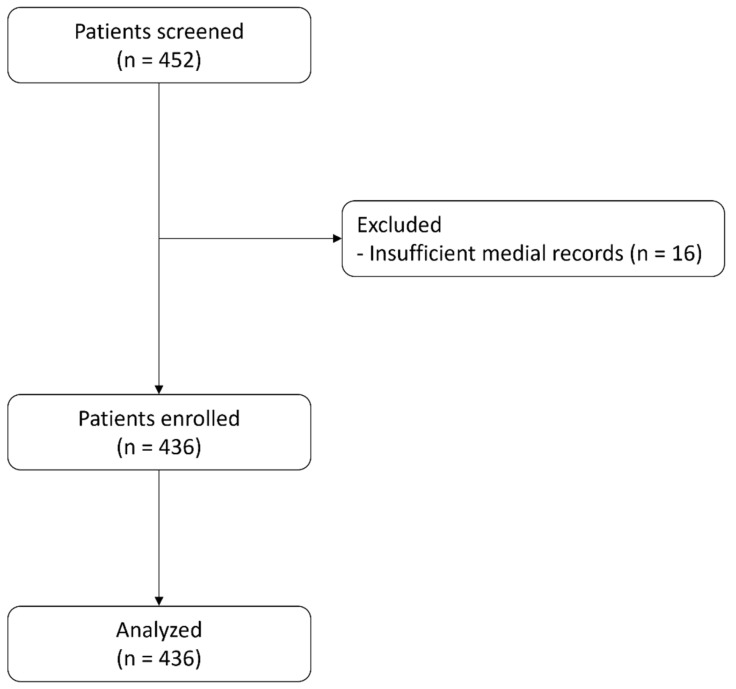
Flow diagram of the study.

A total of 452 patients who underwent CRS-HIPEC from November 2014 to December 2021 were screened for eligibility, from whom 16 patients with insufficient medical records were excluded. Consequently, 436 patients were included in this study (Figure 1). The median follow-up duration was 705 [IQR: 403, 1258] days.

Table 1 summarizes the demographic, comorbidity, and perioperative data categorized according to the CONUT score. A total of 385 patients were classified into the low CONUT score group (score of 0–3), whereas 51 patients were assigned to the high CONUT score group (score of 4–12). Patients in the high CONUT score group were older (59 [50, 66] years vs. 54 [46, 62] years, *p* = 0.022) with higher ASA PS classes (3 [2, 3] vs. 2 [2, 3], *p* < 0.001) and lower BMIs (21.7 [20.2, 23.9] kg/m^2^ vs. 23.1 [20.9, 25.6] kg/m^2^, *p* = 0.020) than those in the low CONUT score group. The prevalence of coronary artery occlusive disease and anemia was higher in the high CONUT score group than in the low CONUT score group (7.8% vs. 1.3%, *p* = 0.013, and 86.3% vs. 39.7%, *p* < 0.001, respectively). The proportion of patients with colorectal cancer was significantly lower in the high CONUT score group than in the low CONUT score group (41.2% vs. 60.8%, *p* = 0.008). No significant differences in intraoperative data were observed between the groups, except for transfused red blood cells. PCI score (18 [8, 39] vs. 14 [5, 26], *p* = 0.022) and CC score (1 [0, 2] vs. 0 [0, 1], *p* = 0.003) were significantly higher in the high CONUT score group than in the low CONUT score group.

Table 2 presents the postoperative outcomes in the CONUT score groups. The length of hospital stay was significantly longer in the high CONUT score group than in the low CONUT score group (16 [12, 23] vs. 14 [11, 18], *p* = 0.032). The incidence of surgical site infection, re-intubation, cardiac complications, and morbidity composite was higher in the high CONUT score group than in the low CONUT score group (39.2% vs. 18.2% for the morbidity composite, *p* < 0.001). The in-hospital mortality rate (7.8% vs. 1.6%, *p* = 0.021) and 1-year mortality rate (47.1% vs. 20.3%, *p* < 0.001) were significantly higher in the high CONUT score group than in the low CONUT score group.

Table 3 shows the results of the logistic regression analysis of the selected variables for predicting 1-year mortality. Multivariable regression analysis revealed that a high CONUT score (OR: 2.253, 95% CI: 1.014–5.005, *p* = 0.046), ASA PS class ≥ 3, history of chronic kidney disease, PCI score ≥ 20, and CC score ≥ 2 were independent risk factors for 1-year mortality. The OR differed depending on pathological characteristics. A simple comparison of variables between the 1-year mortality group and non-mortality groups is shown in Supplementary Table S1. Additionally, the results of the subgroup analysis are summarized in Supplementary Table S2.

Table 4 presents the results of the Cox regression analysis of the selected variables for predicting overall mortality. Multivariable regression analysis indicated that a high CONUT score (HR: 1.777, 95% CI: 1.182–2.669, *p* = 0.006), operative time, PCI score ≥ 20, and CC score ≥ 2 were independent risk factors for overall mortality.

**Figure 2:**
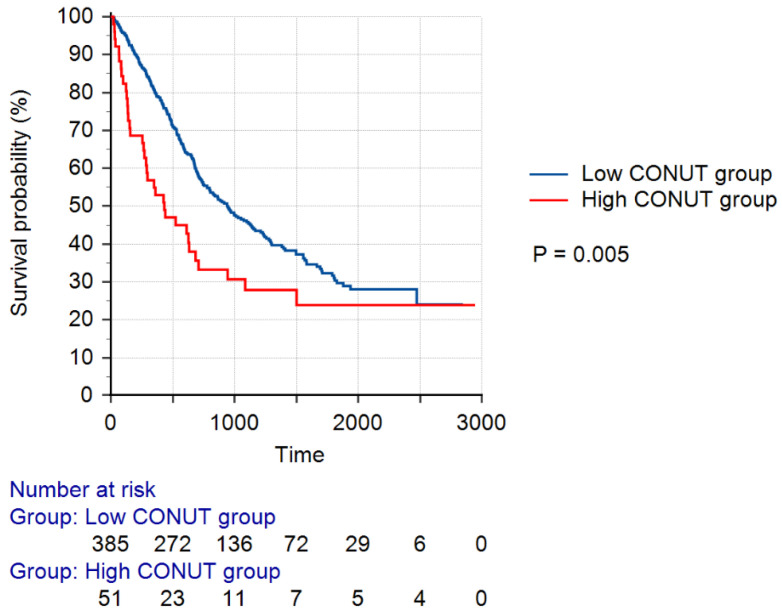
Kaplan–Meier survival curves according to the CONUT score groups. A low CONUT score was defined as a CONUT score of 0–3, whereas a high CONUT score was defined as a CONUT score of 4–12. Abbreviation: CONUT, Controlling Nutritional Status.

Figure 2 shows the Kaplan–Meier survival curves according to the CONUT score groups. The log-rank test indicated that the survival probability of the low CONUT score group was significantly higher than that of the high CONUT score group (*p* = 0.005).

Table 5 presents a logistic regression analysis for morbidity composite outcomes. Multivariable analysis revealed that a high CONUT score (OR: 2.201, 95% CI: 1.066–4.547, *p* = 0.033) and operative time were independent risk factors for the morbidity composite. A simple comparison between groups with and without complications is shown in Supplementary Table S3. The predictive power of other nutritional indicators is summarized in Supplementary Table S4.

## 4. Discussion

To our knowledge, this is the first study that investigated the prognostic value of the CONUT score in patients who underwent CRS-HIPEC. Our findings revealed that patients with malnutrition, as determined by the CONUT score, exhibited significantly higher 1-year mortality and postoperative complication rates than those with normal nutritional status. Notably, a high CONUT score was identified as an independent risk factor of 1-year mortality, overall mortality, and postoperative complications, even after adjustment for confounding factors. These results confirm the potential of the CONUT score as a robust risk stratification tool for identifying high-risk patients within the CRS-HIPEC surgical demographic.

Malnutrition is a recognized risk factor that adversely affects the outcomes of CRS-HIPEC [8,9]; however, there is no consensus on the optimal tool for evaluating nutritional status [7]. Traditional nutritional assessments such as the SGA and serum albumin levels have limitations. The reliability of the SGA depends heavily on the evaluator’s experience and lacks quantitative biochemical measurements [13,14]. Serum albumin level is influenced by various confounding factors such as inflammation, hydration, and kidney function [11,12], and has a long half-life of approximately 19 days [36,37], which limits its utility in monitoring rapid changes in nutritional status. Therefore, there has been recent criticism that albumin is inadequate as a single nutritional indicator. By contrast, the CONUT score, which integrates the lymphocyte count, albumin level, and total cholesterol level, provides a more consistent and comprehensive measure. Prior studies have indicated the superior performance of the CONUT score over albumin alone in predicting outcomes in a variety of patients, including cancer patients [18,38,39,40]. However, its application in CRS-HIPEC has not yet been documented. Our research substantiates the role of the CONUT score as an independent risk factor for mortality and postoperative complications in this context.

The inclusion of cholesterol and lymphocytes in the CONUT score is particularly advantageous because of their role in cancer progression and patient outcomes [41]. Cholesterol level, an integral part of the CONUT score, has been reported to be correlated with the prognosis of cancer patients [42,43]. Moreover, dysregulated cholesterol metabolism has been linked to PI3K/AKT activation and TP53 mutations, which have a close relationship with cancer progression [44,45,46]. In addition, lymphocytes play a pivotal role in the immune response to cancer, with particular types, such as γδ T cells or CD8 T cells, showing a significant influence on cancer progression [47,48,49]. Furthermore, lymphocytopenia has been reported to be associated with the overall survival of cancer patients [50]. On the other hand, the systemic inflammatory response triggered by extensive intraperitoneal chemotherapy and surgical tissue injuries in CRS-HIPEC aggravates malnutrition, highlighting the importance of considering both nutritional and immune statuses in risk stratification and patient management [51,52]. Thus, the comprehensive assessment provided by the CONUT score, encompassing nutritional and immune biomarkers, is invaluable, particularly in the context of CRS-HIPEC, in which patients undergo rigorous treatment protocols.

PCI and CC scores are established prognostic factors in CRS-HIPEC that evaluate the extent of cancer spread and completeness of tumor removal, respectively. These metrics have been consistently validated in numerous studies, and recent meta-analyses have confirmed that the PCI and CC scores are critical indicators of overall survival in patients undergoing CRS-HIPEC [53,54]. Our study reinforces these findings, demonstrating that PCI and CC scores independently predict 1-year and overall mortality. However, despite the clear correlation between these scores and patient survival, their association with postoperative complications appears to be less consistent across different studies [55,56,57]. In our study, while the PCI and CC scores did not significantly predict the morbidity composite, the CONUT score showed substantial predictive power for these complications. This discrepancy suggests that immediate postoperative outcomes are influenced more by the patient’s nutritional status than solely by the extent of cancer progression. Nutrition plays a pivotal role in supporting the immune system [42,43], preventing post-surgical infections [58,59], and facilitating wound healing through collagen synthesis [60,61]. Moreover, maintaining an optimal nutritional status enhances a patient’s tolerance to chemotherapy [62,63], highlighting the importance of nutrition in CRS-HIPEC, which typically involves intensive chemotherapy. Additionally, surgical duration was identified as an independent risk factor for morbidity composite in this study. This observation aligns with previous research indicating that a longer operative time (>240 min) is predictive of major complications after cytoreductive surgery [55]. Extended surgical durations may reflect the complexity of the surgery, which may be influenced by the extent of the procedure or patients’ history of abdominal surgery [55].

The BMI and GNRI, calculated using albumin, body weight, and height, demonstrated significant predictability for one-year mortality in this study. However, they did not show significant results for the morbidity composite. By incorporating lymphocytes and cholesterol in its calculation, the CONUT score exhibited better predictive power than both BMI and GNRI. Lymphocytes and cholesterol can capture aspects of the patient’s condition that albumin alone cannot, indicating that a combination of various biochemical results provides a more comprehensive evaluation of the patient’s nutritional status. Furthermore, our study found that the CONUT and PNI scores exhibited similar predictive power. Previous research [64] has investigated the predictive power of PNI in CRS-HIPEC surgery, reporting that preoperative PNI was a predictor of incomplete cytoreductive surgery. However, there has been no study on the predictive power of the CONUT score in CRS-HIPEC surgery, which is why our study focused on this score. The CONUT score, which has recently gained attention in the field of cancer surgery, theoretically offers more comprehensive information by including cholesterol in its calculation.

While the CONUT score has shown promise as a prognostic tool in this study, it was originally developed to assess nutritional status. It is important to recognize that nutritional assessment and prognostic prediction are distinct, with nutritional status being only one of many factors influencing cancer outcomes. Therefore, a multifactorial approach is essential in risk stratification, and the CONUT score should not be used as a single indicator to predict prognosis. Rather, the CONUT score may be used in conjunction with other risk predictors for risk stratification of patients undergoing CRS-HIPEC.

This study had several limitations that merit consideration. First, the retrospective nature of this study inherently carries the potential for the influence of confounding factors. Despite these challenges, we conducted a multivariable regression analysis to address the issue of confounding variables and enhance the robustness of our findings. Second, this study was performed at a single center, which may have limited the generalizability of the results. Although larger-scale multicenter studies involving more diverse populations might yield different outcomes, it is noteworthy that this study’s sample size of 436 patients was relatively substantial for research on CRS-HIPEC, lending significant weight to our conclusions. Third, our cohort included patients with various types of cancer, each associated with a different prognosis. Although we meticulously categorized and adjusted for pathological characteristics in our regression analyses to mitigate their impact, the diverse nature of the cancers studied suggests that the influence of the CONUT score may vary by cancer type. Future research should explore the specific effects of the CONUT score across different pathological conditions to better understand its prognostic value. Fourth, the cut-off value of CONUT has not yet been firmly established and may vary depending on the disease population. Although our chosen cut-off value aligns with previous studies on cancer surgery [31,32,33,34,35], different cut-off values could potentially produce varying results. Further research is encouraged to refine the optimal cut-off value of the CONUT score. Fifth, three-group analysis may be necessary for a more nuanced and detailed insight into CONUT. However, due to heterogeneity such as cancer type or age variation, a larger sample size is required for post hoc testing based on more detailed multiple group comparisons. We believe that these analyses should be conducted in future larger-scale investigations.

## 5. Conclusions

Patients with CONUT scores ≥ 4 exhibited a significantly higher 1-year mortality rate and postoperative complication rate than those with CONUT scores < 4. This study demonstrated the utility of the CONUT score as an independent predictor of 1-year mortality, overall mortality, and postoperative complications in CRS-HIPEC, underscoring its efficacy in stratifying patient risk and guiding perioperative management in this complex surgical cohort.

## Figures and Tables

**Table 1:** Demographic, comorbidities, and perioperative data according to the CONUT score groups.

	Total	Low CONUT (n = 385)	High CONUT (n = 51)	*p*-Value
Age (years)	55 (46, 62)	54 (46, 62)	59 (50, 66)	0.022
Sex (Female)	231 (53.0%)	205 (53.2%)	26 (51.0%)	0.761
BMI (kg/m^2^)	22.9 (20.8, 25.5)	23.1 (20.9, 25.6)	21.7 (20.2, 23.9)	0.020
ASA PS class	2 (2, 3)	2 (2, 3)	3 (2, 3)	<0.001
Comorbidities				
Hypertension	115 (26.4%)	100 (26.0%)	15 (29.4%)	0.601
DM	57 (13.1%)	52 (13.5%)	5 (9.8%)	0.461
CAOD	9 (2.1%)	5 (1.3%)	4 (7.8%)	0.013
COPD	14 (3.2%)	12 (3.1%)	2 (3.9%)	0.673
Old tuberculosis	14 (3.2%)	13 (3.4%)	1 (2.0%)	>0.999
Hepatitis	12 (2.8%)	10 (2.6%)	2 (3.9%)	0.640
CKD	8 (1.8%)	6 (1.6%)	2 (3.9%)	0.238
Anemia	197 (45.2%)	153 (39.7%)	44 (86.3%)	<0.001
Primary origin				
Colorectal	255 (58.5%)	234 (60.8%)	21 (41.2%)	0.008
Gastric	34 (7.8%)	28 (7.3%)	6 (11.8%)	0.265
Appendiceal/PMP	121 (27.8%)	101 (26.2%)	20 (39.2%)	0.052
Mesothelioma	5 (1.1%)	4 (1.0%)	1 (2.0%)	0.465
Pancreatic	2 (0.5%)	2 (0.5%)	0 (0%)	>0.999
Small bowel	4 (0.9%)	3 (0.8%)	1 (2.0%)	0.393
Others	15 (3.4%)	13 (3.4%)	2 (3.9%)	0.691
Preoperative lab data				
CONUT score	1 (0, 2)	1 (0, 2)	5 (4, 7)	<0.001
Albumin (g/dL)	4.1 (3.8, 4.4)	4.2 (3.9, 4.4)	3.2 (2.9, 3.5)	<0.001
Lymphocyte (/μL)	1655 (1303, 2150)	1740 (1425, 2225)	1020 (760, 1400)	<0.001
Cholesterol (mg/dL)	177 (150, 205)	181 (158, 208)	131 (111, 148)	<0.001
Glucose (mg/dL)	101 (93, 111)	101 (93, 111)	102 (94, 119)	0.394
Creatinine (mg/dL)	0.68 (0.57, 0.85)	0.68 (0.58, 0.85)	0.66 (0.50, 0.83)	0.112
Hemoglobin (g/dL)	12.6 (11.2, 13.8)	12.8 (11.4, 13.9)	10.4 (9.3, 11.4)	<0.001
Intraoperative data				
Operation Time (min)	505 (378, 676)	505 (380, 675)	493 (354, 713)	0.859
Fluid input (mL/h)	739 (633, 859)	743 (640, 856)	697 (625, 888)	0.537
Urine output (mL/h)	115 (77, 162)	115 (79, 162)	100 (63, 162)	0.365
Bleeding (mL)	900 (400, 1600)	900 (400, 1600)	700 (300, 1600)	0.574
Transfused packed RBC (mL)	0 (0, 365)	0 (0, 262)	0 (0, 716)	0.008
PCI score	14 (5, 26)	14 (5, 26)	18 (8, 39)	0.022
CC score	0 (0, 1)	0 (0, 1)	1 (0, 2)	0.003

Values are median (interquartile range) or number (%). A low CONUT score was defined as a CONUT score of 0–3, whereas a high CONUT score was defined as a CONUT score of 4–12. Abbreviations: CONUT, Controlling Nutritional Status; BMI, body mass index; ASA PS class, American Society of Anesthesiologists physical status class; DM, diabetes mellitus; CAOD, coronary artery occlusive disease; COPD, chronic obstructive pulmonary disease; CKD, chronic kidney disease; PMP, Pseudomyxoma peritonei; RBC, red blood cell; PCI, peritoneal cancer index; CC, completeness of cytoreduction.

**Table 2:** Postoperative outcomes according to the CONUT score groups.

	Total	Low CONUT (n = 385)	High CONUT (n = 51)	*p*-Value
ICU length of stay (days)	1 (0, 1)	1 (0, 1)	1 (1, 2)	0.116
Hospital length of stay (days)	14 (11, 19)	14 (11, 18)	16 (12, 23)	0.032
Postoperative complications				
Anastomotic leakage	19 (4.4%)	15 (3.9%)	4 (7.8%)	0.260
Abscess	1 (0.2%)	1 (0.3%)	0 (0%)	>0.999
Gastrointestinal obstruction	8 (1.8%)	6 (1.6%)	2 (3.9%)	0.238
Fistula	4 (0.9%)	3 (0.8%)	1 (2.0%)	0.393
Surgical site infection	15 (3.4%)	10 (2.6%)	5 (9.8%)	0.022
Ascites	29 (6.7%)	24 (6.2%)	5 (9.8%)	0.364
Urinary tract infection	15 (3.4%)	12 (3.1%)	3 (5.9%)	0.401
Pneumonia	19 (4.4%)	14 (3.6%)	5 (9.8%)	0.058
Re-intubation	7 (1.6%)	4 (1.0%)	3 (5.9%)	0.038
Cardiac complication	15 (3.4%)	10 (2.6%)	5 (9.8%)	0.022
Acute kidney injury	14 (3.2%)	12 (3.1%)	2 (3.9%)	0.673
Morbidity composite	90 (20.6%)	70 (18.2%)	20 (39.2%)	<0.001
Reoperation within 30 days	29 (6.7%)	27 (7.0%)	2 (3.9%)	0.557
In-hospital mortality	10 (2.3%)	6 (1.6%)	4 (7.8%)	0.021
1-year mortality	102 (23.4%)	78 (20.3%)	24 (47.1%)	<0.001
Overall mortality	269 (61.7%)	233 (60.5%)	36 (70.6%)	0.165

Values are median (interquartile range) or number (%). A low CONUT score was defined as a CONUT score of 0–3, whereas a high CONUT score was defined as a CONUT score of 4–12. Abbreviations: CONUT, Controlling Nutritional Status; ICU, intensive care unit.

**Table 3:** Logistic regression analysis of chosen variables for predicting 1-year mortality.

Variable	Univariable		Multivariable	
	Crude OR (95% CI)	*p*-Value	Adjusted OR (95% CI)	*p*-Value
High CONUT	3.499 (1.914, 6.397)	<0.001	2.253 (1.014, 5.005)	0.046
Age (years)	1.012 (0.994, 1.030)	0.188		
BMI (kg/m^2^)	0.906 (0.851, 0.965)	0.002	0.948 (0.874, 1.029)	0.204
ASA PS class ≥ 3	2.380 (1.516, 3.737)	<0.001	2.138 (1.199, 3.809)	0.010
CKD	5.687 (1.335, 24.225)	0.019	12.936 (2.190, 76.404)	0.005
Anemia	1.503 (0.963, 2.345)	0.073		
Transfused packed RBC (mL)	1.001 (1.000, 1.001)	0.011	1.000 (1.000, 1.001)	0.193
PCI score ≥ 20	4.402 (2.720, 7.125)	<0.001	2.992 (1.574, 5.688)	0.001
CC score ≥ 2	6.166 (3.738, 10.171)	<0.001	4.757 (2.391, 9.461)	<0.001
Pathology				
Colorectal	Reference		Reference	
Gastric	4.302 (2.058, 8.995)	<0.001	2.783 (1.078, 7.183)	0.034
Appendiceal/PMP	0.518 (0.283, 0.945)	0.032	0.182 (0.083, 0.399)	<0.001
Others	1.798 (0.761, 4.247)	0.181	1.015 (0.309, 3.333)	0.980

A high CONUT score was defined as a CONUT score of 4–12. Abbreviations: OR, odds ratio; CONUT, Controlling Nutritional Status; BMI, body mass index; ASA PS class, American Society of Anesthesiologists physical status class; CKD, chronic kidney disease; RBC, red blood cell; PCI, peritoneal cancer index; CC, completeness of cytoreduction; PMP, Pseudomyxoma peritonei.

**Table 4:** Cox regression analysis of chosen variables for predicting overall mortality.

Variable	Univariable		Multivariable	
	Crude HR (95% CI)	*p*-Value	Adjusted HR (95% CI)	*p*-Value
High CONUT	1.645 (1.157, 2.340)	0.006	1.777 (1.182, 2.669)	0.006
Age (years)	1.000 (0.991, 1.010)	0.998		
Sex (Female)	0.906 (0.713, 1.151)	0.418		
BMI (kg/m^2^)	0.951 (0.919, 0.984)	0.004	0.985 (0.949, 1.022)	0.416
ASA PS class ≥ 3	1.314 (1.031, 1.675)	0.027	1.120 (0.857, 1.463)	0.408
Hypertension	1.073 (0.819, 1.405)	0.611		
DM	0.883 (0.610, 1.277)	0.507		
CAOD	0.839 (0.346, 2.033)	0.697		
COPD	0.446 (0.184, 1.081)	0.074		
Old tuberculosis	0.920 (0.455, 1.860)	0.817		
Hepatitis	0.884 (0.417, 1.872)	0.747		
CKD	1.302 (0.537, 3.160)	0.559		
Anemia	1.340 (1.055, 1.703)	0.017	1.046 (0.801, 1.366)	0.742
Glucose (mg/dL)	1.001 (0.996, 1.006)	0.624		
Creatinine (mg/dL)	1.155 (0.820, 1.629)	0.410		
Operation Time (h)	1.043 (1.006, 1.082)	0.023	1.054 (1.004, 1.106)	0.033
Fluid input (mL/h)	1.000 (0.999, 1.000)	0.297		
Urine output (mL/h)	0.999 (0.997, 1.000)	0.088		
Transfused packed RBC (mL)	1.000 (1.000, 1.001)	0.011	1.000 (1.000, 1.000)	0.790
PCI score ≥ 20	2.200 (1.719, 2.815)	<0.001	1.829 (1.315, 2.543)	<0.001
CC score ≥ 2	2.724 (2.069, 3.586)	<0.001	2.931 (2.039, 4.212)	<0.001
Pathology				
Colorectal	Reference		Reference	
Gastric	2.022 (1.385, 2.951)	<0.001	1.571 (1.038, 2.379)	0.033
Appendiceal/PMP	0.418 (0.298, 0.588)	<0.001	0.227 (0.156, 0.331)	<0.001
Others	1.012 (0.629, 1.629)	0.959	0.477 (0.275, 0.827)	0.008

A high CONUT score was defined as a CONUT score of 4–12. Abbreviations: HR, hazard ratio; CONUT, Controlling Nutritional Status; BMI, body mass index; ASA PS class, American Society of Anesthesiologists physical status class; DM, diabetes mellitus; CAOD, coronary artery occlusive disease; COPD, chronic obstructive pulmonary disease; CKD, chronic kidney disease; RBC, red blood cell; PCI, peritoneal cancer index; CC, completeness of cytoreduction; PMP, Pseudomyxoma peritonei.

**Table 5:** Logistic regression analysis of chosen variables for predicting morbidity composite.

Variable	Univariable		Multivariable	
	Crude OR (95% CI)	*p*-Value	Adjusted OR (95% CI)	*p*-Value
High CONUT	2.903 (1.563, 5.391)	0.001	2.201 (1.066, 4.547)	0.033
Age (years)	1.024 (1.005, 1.044)	0.015	1.015 (0.993, 1.038)	0.171
ASA PS class ≥ 3	2.021 (1.265, 3.228)	0.003	1.438 (0.827, 2.501)	0.198
DM	1.782 (0.956, 3.319)	0.069		
CKD	6.725 (1.576, 28.698)	0.010	1.352 (0.171, 10.671)	0.775
Anemia	1.511 (0.949, 2.407)	0.082		
Creatinine (mg/dL)	3.103 (1.361, 7.077)	0.007	2.632 (0.807, 8.586)	0.109
Operation Time (h)	1.177 (1.099, 1.260)	<0.001	1.130 (1.034, 1.233)	0.007
Transfused packed RBC (mL)	1.001 (1.001, 1.002)	<0.001	1.000 (1.000, 1.001)	0.162
PCI score ≥ 20	1.425 (0.886, 2.290)	0.144		
CC score ≥ 2	1.281 (0.746, 2.201)	0.369		
Pathology				
Colorectal	Reference		Reference	
Gastric	1.152 (0.448, 2.961)	0.769	1.574 (0.584, 4.241)	0.370
Appendiceal/PMP	2.016 (1.194, 3.402)	0.009	1.600 (0.902, 2.838)	0.108
Others	3.942 (1.688, 9.204)	0.002	3.794 (1.507, 9.554)	0.005

A high CONUT score was defined as a CONUT score of 4–12. Abbreviations: OR, odds ratio; CONUT, Controlling Nutritional Status; BMI, body mass index; ASA PS class, American Society of Anesthesiologists physical status class; DM, diabetes mellitus; CKD, chronic kidney disease; RBC, red blood cell; PCI, peritoneal cancer index; CC, completeness of cytoreduction; PMP, Pseudomyxoma peritonei.

## Data Availability

The data presented in this study are available on request from the corresponding author. (The data are not publicly available due to ethical restrictions.)

## Supplementary Materials

The following supporting information can be downloaded at: https://www.mdpi.com/article/10.3390/cancers16152727/s1, Table S1. Demographic, comorbidities, perioperative data according to 1-year mortality; Table S2. Logistic regression analysis of high CONUT scores to predict 1year mortality in subgroup populations; Table S3. Demographic, comorbidities, perioperative data according to morbidity composite; Table S4. Logistic regression analysis of nutritional indicators to predict outcomes after CRS-HIPEC.
