# Supplementary material for: Prognostic Value of the Controlling Nutritional Status (CONUT) Score in Patients Who Underwent Cytoreductive Surgery Combined with Hyperthermic Intraperitoneal Chemotherapy
Source: Cancers (Basel). 2024 Jul 31;16(15):2727. doi: 10.3390/cancers16152727 (PMC11311871; doi:10.3390/cancers16152727)
Supplement: Supplementary file 1 [file cancers-16-02727-s001.zip › cancers-3078763-supplementary.pdf]

Supplementary Table S1. Demographic, comorbidities, perioperative data according to 1-year mortality

| | Non-mortality (n = 334) | 1-year mortality (n = 102) | p-value |
|---|---|---|---|
| Age (years) | 54 (46, 62) | 58 (48, 63) | 0.140 |
| Sex (Female) | 177 (53.0%) | 54 (52.9%) | 0.993 |
| BMI (kg/m²) | 23.1 (21.0, 25.8) | 22.5 (20.1, 24.5) | 0.007 |
| ASA PS class | 2 (2, 3) | 3 (2, 3) | <0.001 |
| Comorbidities | | | |
| Hypertension | 85 (25.4%) | 30 (29.4%) | 0.427 |
| DM | 43 (12.9%) | 14 (13.7%) | 0.823 |
| CAOD | 6 (1.8%) | 3 (2.9%) | 0.443 |
| COPD | 12 (3.6%) | 2 (2.0%) | 0.536 |
| Old tuberculosis | 9 (2.7%) | 5 (4.9%) | 0.332 |
| Hepatitis | 9 (2.7%) | 3 (2.9%) | >0.999 |
| CKD | 3 (0.9%) | 5 (4.9%) | 0.020 |
| Anemia | 143 (42.8%) | 54 (52.9%) | 0.072 |
| Primary origin | | | |
| Colorectal | 197 (59.0%) | 58 (56.9%) | 0.704 |
| Gastric | 15 (4.5%) | 19 (18.6%) | <0.001 |
| Appendiceal, PMP | 105 (31.4%) | 16 (15.7%) | 0.002 |
| Mesothelioma | 3 (0.9%) | 2 (2.0%) | 0.333 |
| Pancreatic | 2 (0.6%) | 0 (0%) | >0.999 |
| Small bowel | 3 (0.9%) | 1 (1.0%) | >0.999 |
| Others | 9 (2.7%) | 6 (5.9%) | 0.128 |
| Preoperative lab data | | | |
| CONUT score | 1 (0, 2) | 1 (0, 3) | 0.046 |
| Albumin (g/dL) | 4.2 (3.9, 4.4) | 4.0 (3.6, 4.2) | <0.001 |
| Lymphocyte (/µl) | 1705 (1390, 2183) | 1550 (1150, 1990) | 0.002 |
| Cholesterol (mg/dL) | 179 ± 41 | 182 ± 44 | 0.468 |
| Glucose (mg/dL) | 100 (93, 110) | 103 (94, 115) | 0.218 |
| Creatinine (mg/dL) | 0.68 (0.57, 0.83) | 0.70 (0.56, 0.89) | 0.355 |
| Hemoglobin (g/dL) | 12.8 (11.3, 13.9) | 12.2 (10.8, 13.2) | 0.007 |
| Intraoperative data | | | |
| Operation Time (min) | 508 (385, 670) | 480 (320, 727) | 0.328 |
| Fluid input (ml/h) | 733 (631, 856) | 766 (641, 880) | 0.514 |
| Urine output (ml/h) | 115 (79, 166) | 115 (67, 154) | 0.319 |
| Bleeding (ml) | 850 (400, 1585) | 1000 (338, 1725) | 0.618 |
| Transfused packed RBC (ml) | 0 (0, 252) | 0 (0, 487) | 0.013 |
| PCI score | 12 (4, 23) | 26 (13, 39) | <0.001 |
| CC score | 0 (0, 1) | 2 (0, 3) | <0.001 |

Values are median (interquartile range) or mean ± standard deviation or number (%). Abbreviations: CONUT, Controlling Nutritional Status; BMI, body mass index; ASA PS class, American Society of Anesthesiologists physical status class, DM, diabetes mellitus; CAOD, coronary artery occlusive disease; COPD, chronic obstructive pulmonary disease; CKD, chronic kidney disease; PMP, Pseudomyxoma peritonei; RBC, red blood cell; PCI, peritoneal cancer index; CC, completeness of cytoreduction.

Supplementary Table S2. Logistic regression analysis of high CONUT scores to predict 1year mortality in subgroup populations.

| Subgroup | Odds ratio (95% CI) | p-value |
|---|---|---|
| CRC (n=255) | 4.377 (1.754, 10.918) | 0.002 |
| Non-CRC (n=181) | 2.960 (1.300, 6.741) | 0.010 |
| PCI ≥ 20 (n=164) | 3.833 (1.531, 9.596) | 0.004 |
| PCI < 20 (n=260) | 3.126 (1.193, 8.193) | 0.020 |
| ASA class ≥ 3 (n=173) | 1.873 (0.883, 3.974) | 0.102 |
| ASA class < 3 (n=263) | 6.517 (2.229, 19.055) | <0.001 |
| Non-Anemia (n=239) | 3.117 (0.674, 14.421) | 0.146 |
| Anemia (n=197) | 3.320 (1.639, 6.726) | <0.001 |
| Non-CKD (n=428) | 3.303 (1.782, 6.121) | <0.001 |
| CKD (n=8) | Not applicable | (-) |

The values represent the odds ratio of high CONUT scores (≥4) for predicting 1-year mortality within subgroups. Abbreviations: CRC; colorectal cancer; PCI, peritoneal cancer index; ASA, American Society of Anesthesiologists; CKD, chronic kidney disease.

Supplementary Table S3. Demographic, comorbidities, perioperative data according to morbidity composite.

| | No complications (n = 346) | Complications (n = 90) | p-value |
|---|---|---|---|
| Age (years) | 53 ± 13 | 57 ± 12 | 0.014 |
| Sex (Female) | 182 (52.6%) | 49 (54.4%) | 0.755 |
| BMI (kg/m$^2$) | 22.9 (20.8, 25.3) | 23.2 (20.4, 26.0) | 0.592 |
| ASA PS class | 2 (2, 3) | 3 (2, 3) | 0.001 |
| Comorbidities | | | |
| Hypertension | 89 (25.7%) | 26 (28.9%) | 0.544 |
| DM | 40 (11.6%) | 17 (18.9%) | 0.066 |
| CAOD | 6 (1.7%) | 3 (3.3%) | 0.400 |
| COPD | 10 (2.9%) | 4 (4.4%) | 0.501 |
| Old tuberculosis | 11 (3.2%) | 3 (3.3%) | >0.999 |
| Hepatitis | 10 (2.9%) | 2 (2.2%) | >0.999 |
| CKD | 3 (0.9%) | 5 (5.6%) | 0.011 |
| Anemia | 149 (43.1%) | 48 (53.3%) | 0.081 |
| Primary origin | | | |
| Colorectal | 215 (62.1%) | 40 (44.4%) | 0.002 |
| Gastric | 28 (8.1%) | 6 (6.7%) | 0.653 |
| Appendiceal, PMP | 88 (25.4%) | 33 (36.7%) | 0.034 |
| Mesothelioma | 3 (0.9%) | 2 (2.2%) | 0.276 |
| Pancreatic | 1 (0.3%) | 1 (1.1%) | 0.371 |
| Small bowel | 2 (0.6%) | 2 (2.2%) | 0.190 |
| Others | 9 (2.6%) | 6 (6.7%) | 0.095 |
| Preoperative lab data | | | |
| CONUT score | 1 (0, 2) | 2 (0, 3) | 0.013 |
| Albumin (g/dL) | 4.2 (3.8, 4.4) | 4.1 (3.5, 4.3) | 0.021 |
| Lymphocyte (/μl) | 1665 (1348, 2150) | 1640 (1230, 2223) | 0.483 |
| Cholesterol (mg/dL) | 179 (152, 206) | 164 (142, 202) | 0.046 |
| Glucose (mg/dL) | 101 (94, 111) | 102 (92, 110) | 0.716 |
| Creatinine (mg/dL) | 0.68 (0.57, 0.84) | 0.72 (0.58, 0.89) | 0.147 |
| Hemoglobin (g/dL) | 12.7 (11.2, 13.7) | 12.1 (11.0, 14.0) | 0.335 |
| Intraoperative data | | | |
| Operation Time (min) | 474 (368, 623) | 633 (430, 789) | <0.001 |
| Fluid input (ml/h) | 749 (632, 860) | 727 (657, 861) | 0.887 |
| Urine output (ml/h) | 115 (77, 162) | 115 (79, 163) | 0.954 |
| Bleeding (ml) | 800 (350, 1400) | 1400 (738, 2175) | <0.001 |
| Transfused packed RBC (ml) | 0 (0, 238) | 249 (0, 709) | <0.001 |
| PCI score | 13 (4, 25) | 18 (8, 31) | 0.010 |
| CC score | 0 (0, 1) | 0 (0, 2) | 0.060 |

Values are median (interquartile range) or mean ± standard deviation or number (%). Abbreviations: CONUT, Controlling Nutritional Status; BMI, body mass index; ASA PS class, American Society of Anesthesiologists physical status class, DM, diabetes mellitus; CAOD, coronary artery occlusive disease; COPD, chronic obstructive pulmonary disease; CKD, chronic kidney disease; PMP, Pseudomyxoma peritonei; RBC, red blood cell; PCI, peritoneal cancer index; CC, completeness of cytoreduction.

Supplementary Table S4. Logistic regression analysis of nutritional indicators to predict outcomes after CRS-HIPEC.

| Variables | 1year mortality | | Morbidity composite | |
|---|---|---|---|---|
| | Odds ratio (95% CI) | p-value | Odds ratio (95% CI) | p-value |
| CONUT | 1.182 (1.057, 1.321) | 0.003 | 1.232 (1.099, 1.381) | <0.001 |
| PNI | 0.921 (0.890, 0.953) | <0.001 | 0.956 (0.924, 0.990) | 0.011 |
| GNRI | 0.945 (0.925, 0.967) | <0.001 | 0.981 (0.960, 1.002) | 0.080 |
| BMI ($kg/m^2$) | 0.906 (0.851, 0.965) | 0.002 | 1.025 (0.964, 1.089) | 0.428 |
| Albumin (g/L) | 0.908 (0.868, 0.949) | <0.001 | 0.927 (0.886, 0.970) | 0.001 |
| Lymphocyte (/μl) | 0.999 (0.999, 1.000) | 0.002 | 1.000 (1.000, 1.000) | 0.812 |
| Cholesterol (mg/dL) | 1.002 (0.997, 1.007) | 0.467 | 0.994 (0.988, 1.000) | 0.052 |

Abbreviations: CONUT, Controlling Nutritional Status; PNI, Prognostic Nutritional Index; GNRI, Geriatric Nutritional Risk Index; BMI, body mass index.
